# Molecular evidence for bacterial pathogens in *Ixodes ricinus* ticks infesting Shetland ponies

**DOI:** 10.1007/s10493-016-0027-4

**Published:** 2016-02-26

**Authors:** Bogumiła Skotarczak, Beata Wodecka, Anna Rymaszewska, Małgorzata Adamska

**Affiliations:** Department of Genetics, University of Szczecin, Felczaka 3c, 71-412 Szczecin, Poland

**Keywords:** *Borrelia*, *Anaplasma*, *Rickettsia*, Questing and feeding *Ixodes ricinus*, PCR, Sequencing

## Abstract

*Ixodes ricinus* has the potential to transmit zoonotic pathogens to humans and domestic animals. The feeding *I. ricinus* (*n* = 1737) collected from 49 Shetland ponies and questing ones from vegetation (*n* = 371) were tested for the presence and differentiation of the bacterial species. DNA of *I. ricinus* ticks was examined with PCR and sequencing analysis to identify species of *Borrelia burgdorferi* sensu lato (*Bb*sl), *Anaplasma phagocytophilum* and *Rickettsia* spp. Altogether, 24.3 % *I. ricinus* of the infested horses and 12.4 % ticks from vegetation carried at least one pathogen species. Horse-feeding ticks (19.2 %) were significantly more frequently infected with *Borrelia* spp. than questing ticks (4.8 %). Among *Bb*sl species, in *I. ricinus* infesting ponies, *B. garinii*, *B. afzelii*, *B. burgdorferi* sensu stricto, *B. valaisiana* and *B. lusitanie* and one species, *B. miyamotoi* related to relapsing fever group, were detected. The 73 *fla*B gene sequences of *Borrelia* obtained from feeding *I. ricinus* have been deposited in GenBank. Among *Rickettsia* species, two were identified: *R. helvetica* which was dominant and *R. monacensis.* Infections with more than one pathogenic species, involving mostly *Bb*sl and *R. helvetica* were detected in 6.3 % of infected ticks collected from horses. Shetland ponies may play an important role in the epidemiological cycle of *Bb*sl and probably could contribute to the natural cycle of *A. phagocytophilum* and *R. helvetica* as host for infected ticks. The awareness about these infectious agents in ticks from ponies might be an important criterion for the risk assessment of human diseases, especially as these animals are maintained for recreational purposes.

## Introduction

In Europe, as well as in Poland, *Ixodes ricinus* is the most common hard tick species and is one of the most important vectors of pathogens for humans and animals (Skotarczak et al. 2003; Wodecka 2003). Among these agents, pathogenic for humans and breeding animals, there are numerous bacteria such as *Borrelia burgdorferi* sensu lato (*Bb*sl), *Anaplasma phagocytophilum* or *Rickettsia* spp. The last two are emerging tick-borne pathogens and cause increasing number of infections in the endemic zones in Europe (Franke et al. 2010).

In *Bb*sl complex of 19 species (Mannelli et al. 2012), some of them are the agents of Lyme borreliosis, the most prevalent tick-borne disease in Europe. Ten species among them and additionally *Borrelia miyamotoi*, related to the relapsing fever spirochetes, have been detected in the European tick vector, *I. ricinus* (Wodecka et al. 2010; Casjens et al. 2011; Lommano et al. 2012).

*Bb*sl infects a wide variety of wild vertebrates, some of which play a role as an infection reservoir but domestic animals like horses are often described as accidental hosts for these pathogens due to the lack of high and persistent bacteremia (Butler et al. 2005; Hansen et al. 2010). In Europe, in humans and dogs, different *B. burgdorferi* species are predisposed to cause different clinical manifestations affecting different organ systems and similar variation in clinical signs of *Bb*sl in infected horses might be due to their infection with different species (Butler et al. 2005; Gall and Pfister 2006). Asymptomatic infections appear to be common in horses as well as in other animal species but description of clinical signs in horses associated with *Bb*sl infections are based on a lower number of case reports (Gall and Pfister 2006). Whether horses may develop clinical disease due to this pathogen, it is controversially discussed, like a role of horses as a competent reservoir.

*Anaplasma phagocytophilum* is the causative agent of granulocytic anaplasmosis occurring in humans and several species of wild and domestic mammals (Rymaszewska and Adamska 2011), including horses (Adaszek and Winiarczyk 2011), and of tick-borne fever in ruminants (Nieder et al. 2012). The first reports in the literature about livestock suffering from a disease called “tick-borne fever” (TBF), have been reported in Europe in 1932 (Gordon et al. 1932), although the history of this fever seems to be even older. A devastating illness of small ruminants was known in western Norway in 1780 (Stuen et al. 2013). Wild ruminants such as roe deer show high infection rates with *A. phagocytophilum* and have been suggested as reservoir hosts (Polin et al. 2004; Skotarczak et al. 2008; Stefanciková et al. 2008). Equine granulocytic ehrlichiosis (*A. phagocytophilum* was formerly known as *Ehrlichia equi*) has been recognized as an infection which causes a wide range of clinical symptoms including lethargy, depression, fever, limb edema and thrombocytopenia (Jahn et al. 2010) but the horses are not known to develop chronic clinical signs (Bakken and Dumler 2000).

The bacteria of the *Rickettsia* genus, obligate intracellular parasites are divided into two subgroups, i.e. the spotted fever group (SFG), composed of almost 20 European species, and the typhus group (TG) with two species only (Rymaszewska and Piotrowski 2013). Rickettsiae are transmitted to vertebrates via hematophagous parasite such as ticks, mites or fleas and they are not only vectors, but also reservoirs for a majority of *Rickettsia* species (Raoult and Roux 1997). Rickettsiae described in Europe belong to the SFG subgroup, the vectors of which are chiefly ticks of the *Ixodes* genus with the leading role of *I. ricinus*. The presence of rickettsiae in that species has been reported in many European countries (Oteo and Portillo 2012).

This study is a part of an application that aimed at better understanding the maintenance of the tick-borne bacteria pathogens in north-western Poland, their endemic region. Earlier, we studied molecular evidence of tick-borne agents in *I. ricinus* collected from vegetation (Skotarczak et al. 2003) and from game animals (Skotarczak et al. 2008). So far, among domestic animals we have examined dogs (Skotarczak et al. 2005; Rymaszewska and Adamska 2011).

In the present paper, to illustrate the diversity of tick-borne pathogens circulating in the domestic host-tick system in the same region of Poland, questing (from vegetation) and feeding on ponies *I. ricinus* ticks were analyzed with molecular methods to determine the presence of the pathogen species with attention to the co-infection of *B. burgdorferi* s.l., *A. phagocytophilum* and *Rickettsia* spp.

## Materials and methods

### Study site and tick collection from ponies and vegetation

Ticks were collected from 49 individuals of Shetland ponies which is a breed of the domestic horse (*Equus caballus*), kept mainly for recreational purposes. The ponies graze on 60 hectare grassland area of Imno Shetlands pony stud at Imno, which is situated on the edge of Goleniowska Forest (north-western Poland, 53°33′20″N 14°56′00″E).

Fed and semi fed ticks were collected in March–June and August–November 2010–2012 from the skin of the ponies permanently kept at the stud. Ticks, all belonging to the *Ixodes ricinus* species, collected from horses (n = 1737) were identified in terms of stage and sex of the imago (females n = 1292, males n = 263, nymphs n = 182, Table 1). In addition, questing *I. ricinus* were collected by flagging vegetation in the area surrounding Imno stud. Among 371 individuals of host-seeking *I. ricinus* collected from the vegetation, there were 6 females, 10 males, 252 nymphs and 103 larvae (Table 3). All ticks were maintained at −20 °C until the DNA extraction.

**Table 1:** Occurrence of DNAs of *Borrelia* spp., *Anaplasma phagocytiphilum* and *Rickettsia* spp. in *Ixodes ricinus* collected from ponies

*I. ricinus*	*I. ricinus*	*Borrelia*	*A. phagocytophilum*	*Rickettsia*
Stages/sex	N/PCR+/%	PCR+/%	PCR+/%	PCR+/%
Females	1292/353/27.3	259/20.0	22/1.7	75/5.8
Males	263/46/17.4	39/14.8	2/0.8	5/1.9
Nymphs	182/48/26.3	35/19.2	2/1.4	11/6.0
Total	1737/450^a^/24.3	333/19.2	26/1.5	91/5.2

^a^450 PCR + *I. ricinus*: 423 individual occurrences and 27 dual co-incidences

### DNA extraction

DNA extraction from ticks was performed with a phenol–chloroform protocol (Wodecka et al. 2014). DNA samples were stored at −70 °C before PCR analyses.

### PCR amplification, species identification and sequencing

All collected *I. ricinus* ticks were examined for the presence of *B. burgdorferi* sensu lato, *A. phagocytophilum* and *Rickettsia* spp. DNA. For this purpose, a screening reaction was conducted using primers amplifying fragments of the *flaB* gene for *Borrelia, msp2* gene for *A. phagocytophilum*, and *gltA* gene for *Rickettsia* spp.

A nested PCR with two primer sets complementary to entire *Borrelia* genus, i.e. outer 132f/905r and inner 220f/823r, was used to detect *flaB* gene fragment of *Borrelia* according to Wodecka et al. (2010). In each PCR run DNA isolated from reference strains of *B. burgdorferi* s.s. IRS, *B. garinii* 20047, *B. afzelii* VS461, *B. valaisiana* VS116, *B. bissettii* DN127 and *B. spielmanii* PC-Eq17 (German Collection of Microorganisms and Cell Cultures—DSMZ, Germany) were used as positive controls and TE buffer as negative control. The PCR products were separated on 1.5 % agarose gel (Bioshop) and archived. The DNA fragments amplified with primers 220f and 823r were digested with enzymes HpyF3I and Ecl136II (Fermentas) to obtain RFLP patterns of different *Borrelia* species according to the protocol by Wodecka (2011).

PCR for *Anaplasma phagocytophilum* and *Rickettsia* sp. employed GoTaq^®^Flexi DNA Polymerase (Promega, USA) at 0.5U/20 μl mix. Final reagent concentrations were 10 mM Tris–HCl (pH 8.3), 50 mM KCl, 1.5 mM MgCl_2_, 200 μM for each deoxynucleoside triphosphate, 10 pM for each primer and 2 μl DNA. PCR conditions were adapted for GoTaq^®^Flexi DNA Polymerase, according to producer’s indications, and the annealing temperatures for respective primer sets were used: *msp2* gene (*A. phagocytophilum*) by Levin et al. (2002), *gltA* gene (*Rickettsia* sp.) by Nilsson et al. (1997). Amplification of the genetic material was carried out in the presence of positive and negative control (without DNA). As positive controls there was used DNA from isolates where previously the presence of the genetic material of bacteria *A. phagocytophilum* or *R. helvetica* had been confirmed. All positive specimens for *Anaplasma* and *Rickettsia* have been sequenced, and the nucleotide sequences were compared to previously obtained and submitted to the GenBank. Analysis of the nucleotide sequences allowed confirming the species belonging of the bacteria detected on the basis of DNA obtained from the ticks’ isolates.

### Tick-borne pathogen DNA sequencing

Partial sequencing of the *flaB* gene fragments of *Borrelia* obtained with primers 220f and 823r was performed for positive samples. These yielded restriction patterns characteristic for different *Borrelia* species. DNA sequencing was performed with dye termination-cycle sequencing. Each strand was analyzed by using an ABI fluorescence automated sequencer.

73 *flaB* gene sequences of *Borrelia* were deposited in the GenBank. The sequences are listed as follows: KR782178–KR782215 (*B. afzelii*), KR782216–KR782218 (*B. burgdorferi* s.s.), KR782219–KR782236 (*B. garinii*), KR782237–KR782249 (*B. lusitanie*), KR782250 (*B. miyamotoi*).

### Statistical analysis

Statistical analysis was performed with a X^2^ test to investigate the differences in infections prevalence between feeding and questing *I. ricinus* and between the stages of ticks. Statistical significance was defined as *P* < 0.05.

## Results

In total, 423 (24.3 %) *I. ricinus* collected from ponies were infected with at least one bacterium (Table 1). *Borrelia* spp. were the most frequently isolated pathogens in ticks, with a total prevalence of 19.2 % (333/1737). Table 1 shows the percentage of infected with *Borrelia* spp. feeding *I. ricinus* ticks, adults and nymphs collected from ponies. Percentages of infected females (20 %; 259/1292) and nymphs (19.2; 35/182) were higher than percentages of males (14.8; 39/263, *P* < 0.005). Among *Bb*sl species in horse-infested *I. ricinus*, 5 were identified and the dominant species was *B. afzelii* (55.2 %; 184/333), then *B. garinii* (29.7 %; 99/333), *B. burgdorferi* sensu stricto (5.4 %; 18/333), *B. valaisiana* (4.5 %; 15/333) and *B. lusitanie* (3.6 %; 12/333, Table 2. Additionally, *B. miyamotoi*, species related to relapsing fever group was identified in 2 ticks (0.6 %; 2/333, Table 2). All recognized species were in females, but 3 in males and nymphs, however both *B. garinii* and *B. afzelii* were in all stages.

**Table 2:** Species of *Borrelia* and *Rickettsia* and co-incidence with *Borrelia* spp., *Anaplasma phagocytophilum* and *Rickettsia helvetica* in *Ixodes ricinus* collected from ponies

*Ixodes ricinus* Stages/sex	*Borrelia* n = 333^a^						*Rickettsia* n = 91		Co-incidence n = 27								
	BG n/%	BA n/%	BB n/%	BV n/%	BL n/%	BM n/%	Rh n/%	Rm n/%	BA/BG n/%	BA/BL n/%	BG/Aph n/%	BA/Aph	BB/Aph	BG/Rh	BA/Rh n/%	BB/Rh	BV/Rh
Females	85/25.5	129/38.7	16/4.8	15/4.5	10/3.0	2/0.6	73/80.2	2/2.2	1/3.7	1/3.7	2/7.4	2/7.4	1/3.7	3/11.1	9/33.3	3/11.1	3/11.1
Males	11/3.3	26/7.8	–	–	2/0.6	–	5/5.5	–	–	–	–	–	–	–	–	–	–
Nymphs	3/0.9	29/8.7	2/0.6	–	–	–	11/12.1	–	1/3.7	–	–	–	–	–	1/3.7	–	–
Total	99/29.7	184/55.2	18/5.4	15/4.5	12/3.6	2/0.6	89/97.8	2/2.2	2/7.4	1/3.7	2/7.4	2/7.4	1/3,7	3/11.1	10/37.0	3/11.1	3/11.1

*BG B. garinii*, *BA* *B. afzelii*, *BB B. burgdorferi* sensu stricto, *BV* *B. valaisiana*, *BL* *B. lusitanie*, *BM B. miyamotoi*, *Aph Anaplasma phagocytophilum*, *Rm Rickettsia monacensis*, *Rh* *Rickettsia helvetica*

^a^333 PCR + *Borrelia*: 330 individual occurrences and 3 dual co-incidences

*Anaplasma phagocytophilum* and *Rickettsia* spp.-infected ticks from ponies had prevalence of 1.5 % (26/1737) and 5.2 % (91/1737), respectively (Table 1). Among *Rickettsia* species, *R. helvetica* constituted 97.8 % and *R. monacensis* 2.2 % of all ticks with rickettsiae (Table 2). Comparing of infected stages showed that females (5.8 %) were significantly more frequently infected with *Rickettsia* than males (1.9 %, *P* < 0.005).

In *I. ricinus* collected from vegetation DNA of *Bb*sl (4.8 %; 18/371), *A. phagocytophilum* (1.3 %; 5/371) and *R. helvetica* (6.2 %; 23/371) was also detected. All infected ticks carried one pathogen (Table 3). Among 6 females one was infected with *Rickettsia* only and among 10 males two with *A. phagocytophilum*, and one of 103 larvae with *Borrelia*, but from 252 nymphs 16.7 % were infected with all 6 species of detected pathogens. Comparison of the stages is difficult as the ratio between different number of tick’s stages from vegetation are diverse from the ones found on ponies excluding nymphs. Comparing the infection rate of the nymphs and performing the statistics showed that feeding nymphs (184/35/19.2 %) were significantly more infected with *Borrelia* spp. than the questing ones (252/17/6.7 %, *P* < 0.005). The similar comparison of the infection rate with *A. phagocytophilum* and *Rickettsia* for total number of ticks’ populations (from ponies and from vegetation) and for nymphs’ populations have not showed significant differences.

**Table 3:** Occurrence and co-incidence of DNAs of *Borrelia* s.l. *Bb*sl (*BG B. garinii*, *BA B. afzelii*, *BB* *B. burgdorferi* sensu stricto, *BL B. lusitaniae*), *Anaplasma phagocytophilum* (Aph) and *Rickettsia helvetica* (Rh) in *Ixodes ricinus* collected from vegetation

Stages/sex	No *I. ricinus*/PCR+ (%)	BbSL	BG PCR+/%	BA PCR+/%	BB PCR+/%	BL PCR+/%	Aph PCR+/%	Rh PCR+/%
Females	6/1 (16.7)	0	0	0	0	0	0	1/16.7
Males	10/2 (20.0)	0	0	0	0	0	2/20	0
Nymphs	252/42 (16.7)	17/6.7	9/3.6	5/2.0	1/0.4	2/0.8	3/1.2	22/8.7
Larvae	103/1 (1.0)	1/1.0	1/1.0	0	0	0	0	0
Total	371/46 (12.4)	18/4.8	10/2.6	5/1.3	1/0.3	2/0.5	5/1.3	23/6.2

Infections with more than one pathogenic species were detected in 27 ticks, which constitutes 6.3 % of infected ticks involving mostly *Bb*sl and *R. helvetica* (Table 2).

## Discussion

Because in some opinion, the molecular assessment of tick-borne pathogens from ticks collected from hosts or vegetation is a suitable way to evaluate the risk of emerging tick-borne diseases in a certain geographical area (Claerebout et al. 2013; Ionita et al. 2013) we examined feeding on ponies and questing *I. ricinus* ticks in the endemic area of Poland. The most frequently identified pathogens were members of the *Bb*sl genospecies complex. The population of *I. ricinus* collected from ponies displayed a high percentage of *Borrelia* infection (19.2 %), higher than in those collected from vegetation (4.8 %, *P* < 0.005) of surrounding meadows where ponies were grazed. The comparing of nymphs’ stages showed that feeding nymphs (184/35/19.2 %) were significantly more frequently infected with *Borrelia* spp. than the questing ones (252/17/6.7 %, *P* < 0.005), but due to the limited sample sizes, *Borrelia* prevalence rates could have not been calculated for other ticks’ stages removed from ponies and from vegetation. The percentage of infection of these questing populations of *I. ricinus* was comparable to the results obtained in the earlier studies of different vegetation sites in north-west Poland (Wodecka 2003; Skotarczak et al. 2008; Wodecka et al. 2010). Such a result, as well as the percentage of the infected adult stages and nymphs of *I. ricinus* ticks fed (or quite-fed) with ponies’ blood could be a suggestion that these ticks obtained the infection from ponies.

Horses are often described as accidental hosts for these pathogens due to the lack of high and persistent bacteremia (Butler et al. 2005; Hansen et al. 2010). However, animals which spend most of their lives out in the open air get a high exposure risk to the tick bites, may obtain infection repeatedly with tick-borne pathogens, especially in grazing horses what gives possibility to spread. Therefore, even though they do not act as a competent reservoir for *Bb*sl because they do not maintain persistent bacteremia, it is evident that these equine hosts may play an important role in the epidemiological cycle of these bacteria.

In Europe, the three most pathogenic species of *B. burgdorferi* s.l. (*Bb*ss, *B. afzelii*, *B. garinii*) are characterized by a different organotropic and pathogenic potential and different species are connected with distinct clinical manifestations of the diseases in humans. So far, very little is known of the pathogenic role of the species of *B. burgdorferi* s.l. in horses because molecular methods are applied for differentiation of *Borrelia* species or strains and they are not commonly used in the veterinary diagnostics (Gall and Pfister, 2006). It is thought that variation in clinical signs of *B. burgdorferi* infecting horses might be due to infection with the same different species similar to those in humans (Butler et al. 2005). DNA of *B. afzelii* in *I. ricinus* ticks feeding on horses was found in Romania (Ionita et al. 2013), DNA of *B. lusitanie* in blood of horses in Lazio, a region of Central Italy (Veronesi et al. 2012). In our studies of *I. ricinus* collected from ponies, among *Bb*sl species, five were detected and the dominant species was *B. afzelii* and *B. garinii*. Furthermore, *B. miyamotoi* related to relapsing fever group was detected in *I. ricinus* collected from the ponies too. Our study confirms the presence of species linked to Lyme borreliosis in *I. ricinus* ticks in Poland and additionally adds new geographic area to the previous findings.

Vector of *A. phagocytophilum* are ticks of *Ixodes* genus, in Europe, they are mainly species belonging to the *I. persulcatus*-complex, i.e. *I. ricinus* and I*. persulcatus* (Paulauskas et al. 2012; Tomanović et al. 2013). The presence of DNA of these bacteria varies within wide limits in European countries.

Wild animals are indicated as a main reservoir for *A. phagocytophilum*, especially wild ruminants, such as white-tailed deer (*Odocoileus virginianus*) in the USA (Johnson et al. 2011) or red deer (*Capreolus capreolus*) and roe deer (*Cervus elaphus*) in Europe (Hulínská et al. 2004; Skotarczak et al. 2008; Stuen et al. 2013). Among domestic animals, small farm ruminants are mostly exposed to *A. phagocytophilum* infection, such as calves, sheep and goats, while the genetic material of *Anaplsma* is rarely detected in the blood of dogs, cats or horses (Stuen et al. 2013). The results of studies of Passamonti et al. (2010) show that horses do not seem to be an appropriate reservoir for *A. phagocytophilum* bacteria.

In Poland, anaplasmosis is a relatively new disease which may cause both therapeutic and diagnostic difficulties in veterinary medicine (Dzięgiel et al. 2013). In Polish studies by Adaszek and Winiarczyk (2011) DNA of *A. phagocytophilum* was found in the blood of horses with clinical symptoms such as movement disorders, fever and debilitation. In our studies of *I. ricinus* collected from ponies, in spite of the high prevalence of ticks on ponies during summer and early autumn months, there is evidence of very little DNA of *A. phagocytophilum*, consistent with our present and earlier studies of *I. ricinus* collected from the vegetation or game animals (Skotarczak et al. 2008), also in north-western part of our country, which showed a low percentage of ticks with DNA of *A. phagocytophilum*. Our result of a low range of occurrence of *A. phagocytophilum* does not eliminate ponies as a reservoir or host but is associated with a low percentage of infection of *I. ricinus* with this bacteria in this area. Thus, ponies probably can contribute to the natural cycle of *A. phagocytophilum*.

In presented studies of ticks collected from ponies, two different *Rickettsia* species were identified. *R. helvetica* was the most common and abundant species and the other species, *R. monacensis* was found in only two females. Whereas in questing *I. ricinus* we identified one species *R. helvetica* only, but prevalence of *Rickettsia* spp. in that two tick populations was parallel (5.2 % from ponies and 6.2 % from vegetation) as well as results of analyzed populations of *I. ricinus,* questing ticks collected from the vegetation from different sites of the same region of Poland (Western Pomerania).

Ticks are a very important link in the life cycle of bacteria of the *Rickettsia* genus. Due to the effective transstadial, transovarial and very rarely described transspermal transmissions, these arachnids can function not only as a vector, but also a reservoir of bacteria (Raoult and Roux 1997; Sprong et al. 2009). The model of circulation of *Rickettsia* sp. including multiplication phase of rickettsiae in the blood of vertebrates has not been confirmed yet.

Overzier et al. (2013) compared the infection of engorged ticks collected from roe deer *C. capreolus* and questing ticks collected from vegetation and they received high results, 16.6 and 13.9 %, respectively, although the differences were not statistically significant. At the same time, the presence of DNA of *Rickettsia* was not detected in blood, spleen and skin of animals, which could testify against the hypothesis indicating vertebrate animals as a potential reservoir of bacteria. Similar results were obtained by Stańczak et al. (2009) from middle-western part of Poland. However, Sprong et al. (2009) suggest that wild animals are involved in the spread of rickettsiae. As a potential reservoir for these bacteria they define a mouse (29 % of *R. helvetica*+), roe deer (19 % of *R. helvetica*+) and wild boar (6.9 % of *R. helvetica*+), while no *Rickettsia* DNA was found in blood of red deer, although the *I. ricinus* often feeds on this species. The results obtained by Sprong et al. (2009) may confirm the participation of some vertebrates in the life cycle of some species of *Rickettsia* spp., at least for a short period of time (short-term bacteremia).

In Europe, the prevalence of tick carrying multiple pathogens has been reported by many studies. For the reason that mixed infections (including species of same genus) and coinfections (including species of different genera) are of medical importance, it is crucial to determine the prevalence of ticks infected by more than one pathogen. In our earlier studies of ticks collected from the vegetation we showed for the first time in our country a double and triple co-incidence of tick-borne pathogens in *I. ricinus* (Skotarczak et al. 2003). Later, co-infections with tick-borne agents were observed in *I. ricinus* from game animals or in tissues of these animals (Skotarczak et al. 2008) and of dogs (Skotarczak and Wodecka 2003). In horses, the occurrence and characteristics of co-infections with multiple tick-borne agents have not been documented yet, but they seem to be common in some parts of the world (Hansen et al. 2010; Ybañez et al. 2013). In the study of Laus et al. (2013) blood samples from 300 Italian horses were analyzed for the presence of antibodies against tick-borne agents and their DNA and 24 animals were found to have a double infection, 2 triple, but no special clinical signs were observed in any of these animals. In our present studies of feeding *I. ricinus* collected from ponies, infections with more than one pathogenic species, involving mostly *Bb*sl and *R. helvetica* were detected in only 6.3 % of all infected ticks. However, it must be taken into account for risk assessment of human diseases, especially as these animals are maintained for recreational purposes.

## Conclusions

The results of the present study confirm the presence of very diverse bacterial pathogens at the domestic host-tick interface, with the potential to cause both animal and human disease. A higher percentage of fed with blood ticks infected with *Bb*sl than questing ones suggests that an infection was acquired from horses and ticks are a composition in the epidemiological cycle of *Bb*sl. Probably ponies also could contribute to the natural cycle of *A. phagocytophilum* and *R. helvetica*.

The cognizance of the prevalence of these infectious agents in ticks collected from ponies is an important criterion for risk assessment of human diseases, especially as these animals are maintained for recreational purposes and lots of ticks are present on them. Additionally, the results suggest that there may be accidental simultaneous double transmission of tick-borne pathogens to human beings. This is the first study reporting tick-borne pathogens prevalence in ticks removed from horses in Poland.
